# *N*-(4-Chloro­phen­yl)-1-(5-{[(2-phenyl­ethen­yl)sulfon­yl]meth­yl}-1,3,4-oxadiazol-2-yl)methane­sulfonamide

**DOI:** 10.1107/S1600536812037300

**Published:** 2012-09-19

**Authors:** A. Muralikrishna, M. Kannan, V. Padmavathi, A. Padmaja, R. Krishna

**Affiliations:** a Department of Chemistry, Sri Venkateswara University, Tirupati 517 502, India; b Centre for Bioinformatics, Pondicherry University, Puducherry 605 014, India

## Abstract

In the title compound, C_18_H_16_ClN_3_O_5_S_2_, the dihedral angles between the oxadiazole ring and the phenyl and chloro­benzene rings are 23.4 (2) and 45.4 (2)°, respectively. The C—S—N—C and C_ox_—C—S—C (ox = oxadiazole) torsion angles are 89.3 (5) and −69.1 (3)°, respectively. A short intra­molecular C—H⋯O contact closes an *S*(6) ring. In the crystal, mol­ecules are linked by N—H⋯O hydrogen bonds, generating *C*(10) chains propagating in [001]. The packing is consolidated by C—H⋯O, C—H⋯π and very weak aromatic π–π stacking inter­actions [centroid–centroid separation = 4.085 (2) Å].

## Related literature

For the synthesis and biological activity of the title compound, see: Padmaja *et al.* (2011 ▶); Muralikrishna *et al.* (2012 ▶). For related structures, see: Ranjith *et al.* (2009 ▶); You *et al.* (2004 ▶).
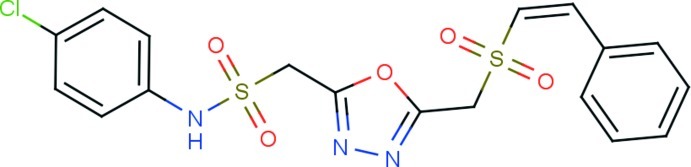


## Experimental

### 

#### Crystal data

C_18_H_16_ClN_3_O_5_S_2_
*M* *_r_* = 453.93
Monoclinic, 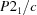

*a* = 21.1387 (12) Å
*b* = 5.4443 (2) Å
*c* = 18.3484 (11) Å
β = 107.810 (7)°
*V* = 2010.4 (2) Å^3^
*Z* = 4
Mo *K*α radiation
μ = 0.43 mm^−1^
*T* = 293 K
0.20 × 0.20 × 0.06 mm

#### Data collection

Oxford Diffraction Xcalibur Eos diffractometer
Absorption correction: multi-scan (*CrysAlis PRO*; Oxford Diffraction, 2009 ▶) *T*_min_ = 0.917, *T*_max_ = 0.974
8939 measured reflections
3541 independent reflections
2114 reflections with *I* > 2σ(*I*)
*R*_int_ = 0.052

#### Refinement

*R*[*F* ^2^ > 2σ(*F* ^2^)] = 0.051
*wR*(*F* ^2^) = 0.171
*S* = 0.87
3541 reflections
262 parameters
H-atom parameters constrained
Δρ_max_ = 0.26 e Å^−3^
Δρ_min_ = −0.29 e Å^−3^

### 

Data collection: *CrysAlis CCD* (Oxford Diffraction, 2009 ▶); cell refinement: *CrysAlis CCD*; data reduction: *CrysAlis RED* (Oxford Diffraction, 2009 ▶); program(s) used to solve structure: *SHELXS97* (Sheldrick, 2008 ▶); program(s) used to refine structure: *SHELXL97* (Sheldrick, 2008 ▶); molecular graphics: *ORTEP-3* (Farrugia, 1997 ▶); software used to prepare material for publication: *PLATON* (Spek, 2009 ▶).

## Supplementary Material

Crystal structure: contains datablock(s) I, global. DOI: 10.1107/S1600536812037300/hb6934sup1.cif

Structure factors: contains datablock(s) I. DOI: 10.1107/S1600536812037300/hb6934Isup2.hkl

Supplementary material file. DOI: 10.1107/S1600536812037300/hb6934Isup3.cml

Additional supplementary materials:  crystallographic information; 3D view; checkCIF report

## Figures and Tables

**Table 1:** Hydrogen-bond geometry (Å, °) *Cg* is the centroid of the C4–C9 ring.

*D*—H⋯*A*	*D*—H	H⋯*A*	*D*⋯*A*	*D*—H⋯*A*
C5—H5⋯O3	0.93	2.41	3.010 (6)	122
N3—H3⋯O5^i^	0.86	2.19	2.900 (5)	140
C3—H3*B*⋯O2^ii^	0.97	2.38	3.198 (5)	141
C6—H6⋯O4^iii^	0.93	2.45	3.290 (5)	151
C12—H12⋯O5^iii^	0.93	2.60	3.242 (5)	127
C14—H14⋯*Cg* ^iv^	0.93	2.90	3.670 (5)	141

Symmetry codes: (i) 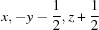; (ii) 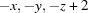; (iii) 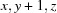; (iv) 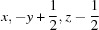.

**Fig. 1.**
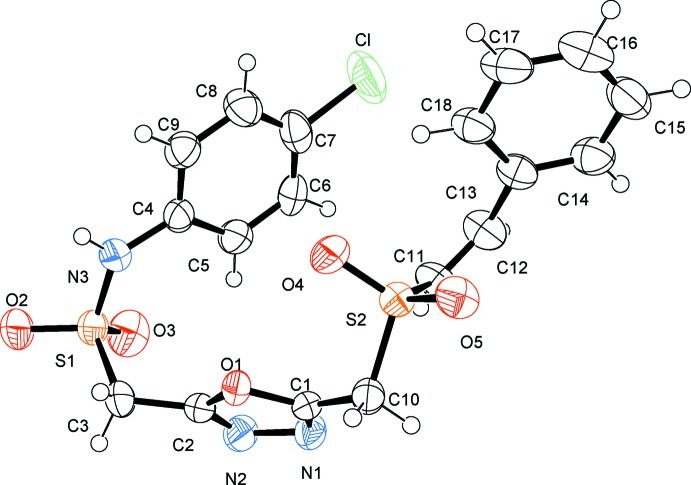
: The molecular structure of (I), showing displacement ellipsoids drawn at the 50% probability level.

**Fig. 2.**
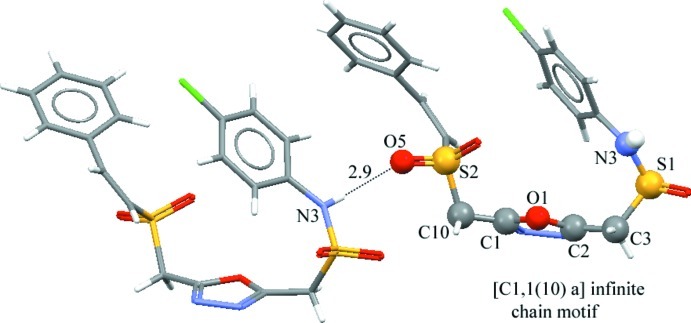
: A view of C11 (10) infinite chain motif is formed between N3—H3···.O5 with the hydrogen bond symmetry equivalent. Infinite chain motif forming atoms are shown in ball and stick model and the hydrogen bond is shown in black dashed line.

**Fig. 3.**
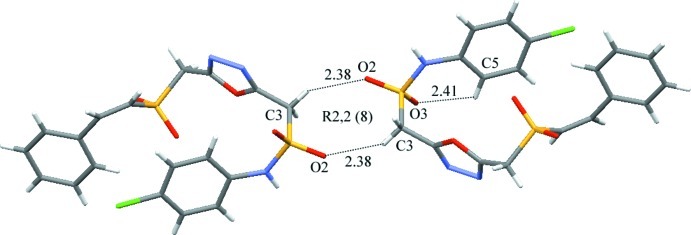
: A view of R22 (8) ring motifs formed by C3—H3B···O2 interaction between to molecules. The C5—H5···.O3 forms an intramolecuar interaction. The Hydrogen bonds are shown in black dashed lines.

**Fig. 4.**
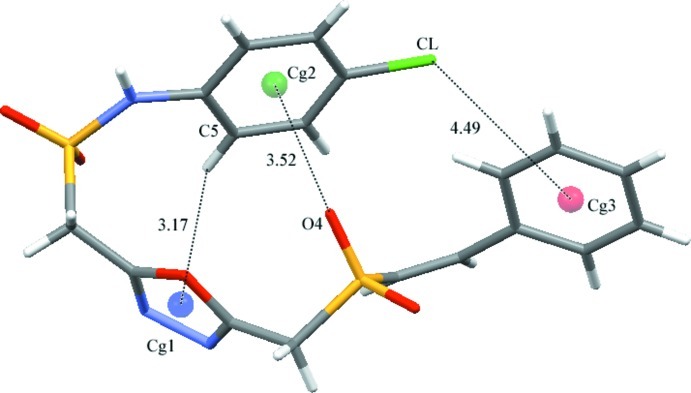
: The special type of intramolecular interaction is formed between C5—H5···.pi (Cg1: O1, C1, N1, N2 & C2), S2—O4···.pi (Cg2: C4, C5, C6, C7, C8 & C9) and C7—CL···.pi (Cg3: C13, C14, C15, C16, C17 & C18) with a distance of 3.17, 3.52 and 4.49 Å respectively. The centroids are shown in different color with corresponding labeling.

**Fig. 5.**
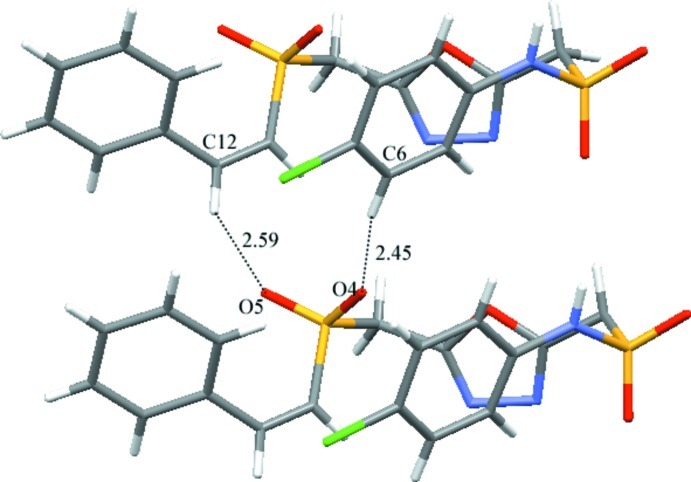
: The intermolecular C12—H12···.O5 and C6—H6···.O4 interaction are shown.

**Fig. 6.**
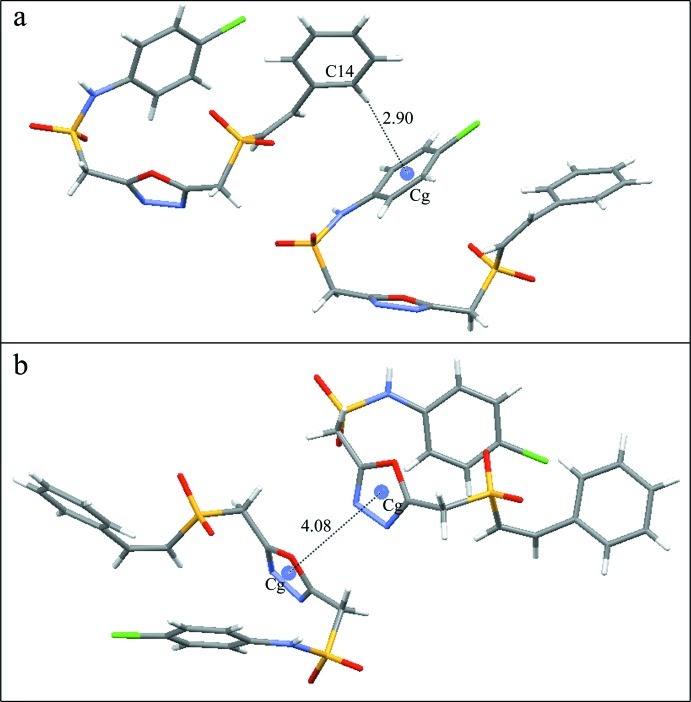
: a) The molecular interaction showing the C—H···pi interaction between two molecules, in which the Cg is the centriod of C4—C9 ring. b) The pi-pi stacking interaction also shown between the oxadiazole ring. The contacts distance are shown in black dashed lines.

## supplementary crystallographic information

### Comment

The title compound, (I), is a sulfone linked bis-heterocyclic and it has antimicrobial and cytotoxicity activity (Padmaja *et al.*, 2011; Muralikrishna *et al.*, 2012). As part of our ongoing studies on this compound, we now describe its crystal structure.

In the title compound (I) the phenylethenesulfonyl moiety deviates significantly from the plane of dimethyl oxadiazole ring by an (+)-*anti*-periplanar conformation with the torsion angle (C_10_, S_2_, C_11_ & C_12_) of 155.0 (5)°. In case of Chlorophenylaminosulfonyl moiety attached with dimethyl oxadiazole and deviates from the plane by an (-)-*syn*-clinal conformation with the torsion angle (C_3_, S_1_, N_3_ & C_4_) of 89.3 (5)°. The plane of oxadiazole ring intersect bisectionally to the cholorophenyl ring plane with angle of 45.4 (2) °, whereas it axially intersect with phenyl ring plane by the angle of 23.4 (2) ° (Fig. 1). The strong intermolecular hydrogen bond is formed between N_3_—H_3_···.O_5_ with a distance of 2.900 (5) Å, which generates a C_1_^1^(10) infinite chain motif (Ranjith *et al.*, 2009) with the hydrogen bond symmetry equivalent (Fig.2). The intermolecular C_3_—H_3_B···.O_2_ makes *R*_2_^2^ (8) motif between the adjacent molecules by the contact distance of 3.198 (5) Å and shown in Fig. 3. The intramolecular interaction is formed by C_5_—H_5_···.O_3_ with a distance of 3.010 (6) Å (Fig. 3). In addition to that, the special type of intramolecular interaction also formed between C_5_—H_5_···.π (*Cg*1: O_1_, C_1_, N_1_, N_2_ & C_2_), S_2_—O_4_···.π (*Cg*2: C_4_, C_5_, C_6_,*C*_7_, C_8_ & C_9_) and C_7_—CL···.π (*Cg*3: C_13_, C_14_, C_15_, C_16_, C_17_ & C_18_) with a contact distance of 3.17, 3.52 and 4.49 Å respectively (Fig. 4), which contributes for the intramolecular packing. In addition to the aforementioned intermolecular interaction, the C_6_—H_6_···.O_4_ and C_12_—H_12_···.O_5_ makes short contact with the distance of 3.290 (5) and 3.242 (5) Å respectively (Fig. 5). Moreover, the intermolecular C_14_—H_14_···.π (*Cg*: C_4_, C_5_, C_6_,*C*_7_ & C_8_) and π-π (*Cg*: O_1_, C_1_, N_1_,*N*_2_ & C_2_) stacking interaction (You *et al.*, 2004) is formed by the distance of 3.670 (5) and 4.085 (2) Å respectively (Fig. 6a & b).

### Experimental

A mixture of *p*-chlorophenylaminosulfonylacetic acid hydrazide (10 mmol), *Z*-styrylsulfonylacetic acid (10 mmol) and POCl_3_ (7 ml) was heated under reflux for 5–7 h. After completion of the reaction (monitored by TLC), the reaction mixture was cooled, the excess POCl_3_ was removed under reduced pressure and the residue was poured onto crushed ice. The resulting precipitate was filtered, washed with saturated sodium bicarbonate solution and then with water to give 2-(*p*-Chlorophenylaminosulfonyl-methyl)-5-[*Z*-(styrylsulfonylmethyl)]-1,3,4-oxadiazole (69%, m.p. = 134–136 °C). Colourless plates were recrystallized from a methanol–dichloromethane (10:1) solution.

### Refinement

The non-hydrogen atoms where refined anisotropically whereas hydrogen atoms were refined isotropically. The H atoms were geometrically placed (N—H = 0.86 Å, and C—H=0.93–0.97 Å) and refined as riding with *U*_iso_ (H) = 1.2–1.5 *U*_eq_ (parent atom).

### Crystal data

**Table:**

C_18_H_16_ClN_3_O_5_S_2_	*F*(000) = 936
*M**_r_* = 453.93	*D*_x_ = 1.513 Mg m^−^^3^
Monoclinic, *P*2_1_/*c*	Mo *K*α radiation, λ = 0.7107 Å
Hall symbol: -P 2ybc	Cell parameters from 2041 reflections
*a* = 21.1387 (12) Å	θ = 2.6–29.2°
*b* = 5.4443 (2) Å	µ = 0.43 mm^−^^1^
*c* = 18.3484 (11) Å	*T* = 293 K
β = 107.810 (7)°	Plate, colourless
*V* = 2010.4 (2) Å^3^	0.20 × 0.20 × 0.06 mm
*Z* = 4	

### Data collection

**Table:**

Oxford Diffraction Xcalibur Eos diffractometer	3541 independent reflections
Radiation source: Enhance (Mo) X-ray Source	2114 reflections with *I* > 2σ(*I*)
Graphite monochromator	*R*_int_ = 0.052
Detector resolution: 15.9821 pixels mm^-1^	θ_max_ = 25.0°, θ_min_ = 2.6°
ω scans	*h* = −25→22
Absorption correction: multi-scan (*CrysAlis PRO*; Oxford Diffraction, 2009)	*k* = −6→6
*T*_min_ = 0.917, *T*_max_ = 0.974	*l* = −2→21
8939 measured reflections	

### Refinement

**Table:**

Refinement on *F*^2^	Primary atom site location: structure-invariant direct methods
Least-squares matrix: full	Secondary atom site location: difference Fourier map
*R*[*F*^2^ > 2σ(*F*^2^)] = 0.051	Hydrogen site location: inferred from neighbouring sites
*wR*(*F*^2^) = 0.171	H-atom parameters constrained
*S* = 0.87	*w* = 1/[σ^2^(*F*_o_^2^) + (0.1*P*)^2^] where *P* = (*F*_o_^2^ + 2*F*_c_^2^)/3
3541 reflections	(Δ/σ)_max_ < 0.001
262 parameters	Δρ_max_ = 0.26 e Å^−^^3^
0 restraints	Δρ_min_ = −0.29 e Å^−^^3^

### Special details

**Table:**

Geometry. All e.s.d.'s (except the e.s.d. in the dihedral angle between two l.s. planes) are estimated using the full covariance matrix. The cell e.s.d.'s are taken into account individually in the estimation of e.s.d.'s in distances, angles and torsion angles; correlations between e.s.d.'s in cell parameters are only used when they are defined by crystal symmetry. An approximate (isotropic) treatment of cell e.s.d.'s is used for estimating e.s.d.'s involving l.s. planes.
Refinement. Refinement of *F*^2^ against ALL reflections. The weighted *R*-factor *wR* and goodness of fit *S* are based on *F*^2^, conventional *R*-factors *R* are based on *F*, with *F* set to zero for negative *F*^2^. The threshold expression of *F*^2^ > σ(*F*^2^) is used only for calculating *R*-factors(gt) *etc*. and is not relevant to the choice of reflections for refinement. *R*-factors based on *F*^2^ are statistically about twice as large as those based on *F*, and *R*- factors based on ALL data will be even larger.

### Fractional atomic coordinates and isotropic or equivalent isotropic displacement parameters (Å^2^)

**Table:**

	*x*	*y*	*z*	*U*_iso_*/*U*_eq_	
S1	0.10737 (6)	0.1465 (2)	0.99341 (7)	0.0506 (4)	
S2	0.20592 (5)	−0.11531 (16)	0.70760 (6)	0.0365 (3)	
Cl	0.40975 (8)	0.2776 (3)	0.90563 (11)	0.0990 (6)	
O1	0.09206 (13)	−0.0641 (5)	0.80065 (15)	0.0409 (7)	
O5	0.21845 (15)	−0.2584 (5)	0.64804 (18)	0.0506 (8)	
O4	0.22585 (15)	−0.2151 (5)	0.78332 (18)	0.0558 (9)	
O2	0.08523 (16)	0.0536 (7)	1.05419 (19)	0.0720 (10)	
N1	0.07298 (17)	0.2839 (6)	0.7363 (2)	0.0450 (9)	
C1	0.09493 (18)	0.0632 (7)	0.7380 (2)	0.0348 (9)	
O3	0.11247 (18)	0.4033 (6)	0.9846 (2)	0.0719 (10)	
N3	0.17730 (17)	0.0164 (7)	1.0028 (2)	0.0574 (11)	
H3	0.1836	−0.1171	1.0291	0.069*	
N2	0.05241 (18)	0.3095 (7)	0.8019 (2)	0.0500 (10)	
C10	0.11833 (19)	−0.0673 (7)	0.6803 (2)	0.0403 (10)	
H10A	0.1050	0.0260	0.6331	0.048*	
H10B	0.0963	−0.2256	0.6701	0.048*	
C13	0.3483 (2)	0.0561 (7)	0.6906 (3)	0.0454 (11)	
C14	0.3805 (2)	0.0969 (8)	0.6365 (3)	0.0577 (13)	
H14	0.3684	0.2316	0.6040	0.069*	
C7	0.3391 (2)	0.1971 (9)	0.9301 (3)	0.0568 (13)	
C6	0.2849 (3)	0.3435 (8)	0.9081 (3)	0.0530 (12)	
H6	0.2847	0.4818	0.8783	0.064*	
C8	0.3399 (2)	−0.0127 (9)	0.9717 (3)	0.0557 (13)	
H8	0.3767	−0.1158	0.9839	0.067*	
C9	0.2858 (2)	−0.0691 (8)	0.9950 (3)	0.0496 (11)	
H9	0.2863	−0.2097	1.0239	0.059*	
C18	0.3694 (2)	−0.1416 (8)	0.7403 (3)	0.0526 (13)	
H18	0.3497	−0.1715	0.7783	0.063*	
C3	0.0515 (2)	0.0242 (9)	0.9082 (2)	0.0534 (12)	
H3A	0.0535	−0.1537	0.9112	0.064*	
H3B	0.0067	0.0730	0.9054	0.064*	
C12	0.2957 (2)	0.2238 (7)	0.6963 (3)	0.0539 (13)	
H12	0.3047	0.3894	0.6921	0.065*	
C11	0.2379 (2)	0.1785 (7)	0.7066 (3)	0.0464 (12)	
H11	0.2127	0.3112	0.7137	0.056*	
C5	0.2297 (2)	0.2862 (8)	0.9301 (3)	0.0513 (12)	
H5	0.1920	0.3848	0.9144	0.062*	
C2	0.06481 (19)	0.1027 (8)	0.8369 (2)	0.0412 (10)	
C4	0.2303 (2)	0.0831 (7)	0.9755 (2)	0.0442 (11)	
C17	0.4193 (2)	−0.2922 (9)	0.7334 (3)	0.0633 (15)	
H17	0.4330	−0.4230	0.7671	0.076*	
C15	0.4302 (2)	−0.0575 (10)	0.6295 (3)	0.0667 (15)	
H15	0.4507	−0.0283	0.5921	0.080*	
C16	0.4491 (2)	−0.2543 (9)	0.6781 (4)	0.0674 (16)	
H16	0.4820	−0.3610	0.6733	0.081*	

### Atomic displacement parameters (Å^2^)

**Table:**

	*U*^11^	*U*^22^	*U*^33^	*U*^12^	*U*^13^	*U*^23^
S1	0.0458 (7)	0.0765 (9)	0.0334 (7)	0.0029 (6)	0.0177 (5)	−0.0018 (6)
S2	0.0403 (6)	0.0290 (5)	0.0402 (7)	0.0032 (4)	0.0122 (5)	−0.0008 (5)
Cl	0.0756 (11)	0.1391 (14)	0.1018 (13)	−0.0272 (9)	0.0562 (10)	−0.0005 (10)
O1	0.0424 (17)	0.0491 (16)	0.0336 (17)	0.0029 (13)	0.0153 (14)	0.0082 (13)
O5	0.0518 (19)	0.0459 (16)	0.056 (2)	0.0031 (13)	0.0189 (16)	−0.0183 (14)
O4	0.053 (2)	0.0610 (18)	0.050 (2)	0.0075 (14)	0.0103 (16)	0.0195 (16)
O2	0.059 (2)	0.123 (3)	0.042 (2)	0.000 (2)	0.0281 (17)	0.003 (2)
N1	0.048 (2)	0.050 (2)	0.040 (2)	0.0115 (17)	0.0184 (18)	0.0058 (17)
C1	0.030 (2)	0.043 (2)	0.029 (2)	−0.0014 (17)	0.0059 (18)	0.0037 (18)
O3	0.091 (3)	0.063 (2)	0.066 (3)	0.0071 (18)	0.032 (2)	−0.0110 (17)
N3	0.039 (2)	0.078 (3)	0.057 (3)	0.0039 (19)	0.0164 (19)	0.027 (2)
N2	0.051 (2)	0.060 (2)	0.042 (2)	0.0137 (18)	0.0184 (19)	−0.0010 (19)
C10	0.043 (3)	0.043 (2)	0.037 (3)	0.0001 (19)	0.015 (2)	0.0014 (19)
C13	0.039 (3)	0.038 (2)	0.059 (3)	−0.0046 (19)	0.014 (2)	−0.004 (2)
C14	0.050 (3)	0.054 (3)	0.067 (4)	0.001 (2)	0.014 (3)	0.013 (2)
C7	0.060 (3)	0.070 (3)	0.048 (3)	−0.019 (3)	0.027 (3)	−0.014 (3)
C6	0.069 (4)	0.052 (3)	0.042 (3)	−0.006 (2)	0.023 (3)	0.008 (2)
C8	0.050 (3)	0.063 (3)	0.057 (3)	0.006 (2)	0.022 (3)	−0.005 (3)
C9	0.053 (3)	0.048 (2)	0.048 (3)	0.005 (2)	0.016 (2)	0.007 (2)
C18	0.039 (3)	0.049 (3)	0.069 (4)	−0.001 (2)	0.015 (2)	0.007 (2)
C3	0.044 (3)	0.083 (3)	0.037 (3)	−0.005 (2)	0.020 (2)	0.002 (2)
C12	0.051 (3)	0.034 (2)	0.080 (4)	0.002 (2)	0.026 (3)	0.006 (2)
C11	0.042 (3)	0.032 (2)	0.069 (3)	0.0069 (18)	0.023 (2)	−0.003 (2)
C5	0.047 (3)	0.053 (3)	0.053 (3)	0.000 (2)	0.013 (2)	0.013 (2)
C2	0.031 (2)	0.059 (3)	0.034 (3)	0.0007 (19)	0.0091 (19)	0.001 (2)
C4	0.041 (3)	0.057 (3)	0.033 (3)	−0.009 (2)	0.010 (2)	0.000 (2)
C17	0.039 (3)	0.057 (3)	0.087 (4)	0.005 (2)	0.010 (3)	0.015 (3)
C15	0.046 (3)	0.085 (4)	0.075 (4)	−0.003 (3)	0.027 (3)	−0.010 (3)
C16	0.043 (3)	0.061 (3)	0.096 (5)	0.002 (2)	0.018 (3)	−0.013 (3)

### Geometric parameters (Å, º)

**Table:**

S1—O3	1.415 (3)	C7—C6	1.353 (7)
S1—O2	1.428 (3)	C7—C8	1.370 (7)
S1—N3	1.600 (4)	C6—C5	1.383 (6)
S1—C3	1.775 (5)	C6—H6	0.9300
S2—O4	1.430 (3)	C8—C9	1.373 (6)
S2—O5	1.431 (3)	C8—H8	0.9300
S2—C11	1.739 (4)	C9—C4	1.391 (6)
S2—C10	1.783 (4)	C9—H9	0.9300
Cl—C7	1.741 (5)	C18—C17	1.371 (6)
O1—C2	1.354 (5)	C18—H18	0.9300
O1—C1	1.359 (4)	C3—C2	1.484 (6)
N1—C1	1.285 (5)	C3—H3A	0.9700
N1—N2	1.406 (5)	C3—H3B	0.9700
C1—C10	1.480 (5)	C12—C11	1.315 (6)
N3—C4	1.409 (5)	C12—H12	0.9300
N3—H3	0.8600	C11—H11	0.9300
N2—C2	1.283 (5)	C5—C4	1.381 (6)
C10—H10A	0.9700	C5—H5	0.9300
C10—H10B	0.9700	C17—C16	1.365 (7)
C13—C14	1.382 (6)	C17—H17	0.9300
C13—C18	1.393 (6)	C15—C16	1.373 (7)
C13—C12	1.468 (6)	C15—H15	0.9300
C14—C15	1.382 (6)	C16—H16	0.9300
C14—H14	0.9300		
			
O3—S1—O2	119.6 (2)	C5—C6—H6	120.2
O3—S1—O2	119.6 (2)	C7—C8—C9	119.4 (4)
O3—S1—N3	110.4 (2)	C7—C8—H8	120.3
O3—S1—N3	110.4 (2)	C9—C8—H8	120.3
O2—S1—N3	105.8 (2)	C8—C9—C4	120.3 (4)
O3—S1—C3	108.9 (2)	C8—C9—H9	119.9
O3—S1—C3	108.9 (2)	C4—C9—H9	119.9
O2—S1—C3	105.4 (2)	C17—C18—C13	120.1 (5)
N3—S1—C3	105.8 (2)	C17—C18—H18	119.9
O4—S2—O5	117.81 (18)	C13—C18—H18	119.9
O4—S2—C11	111.1 (2)	C2—C3—S1	114.5 (3)
O5—S2—C11	109.2 (2)	C2—C3—H3A	108.6
O4—S2—C10	107.48 (19)	S1—C3—H3A	108.6
O5—S2—C10	106.52 (18)	C2—C3—H3B	108.6
C11—S2—C10	103.63 (19)	S1—C3—H3B	108.6
C2—O1—C1	102.1 (3)	H3A—C3—H3B	107.6
C1—N1—N2	106.0 (3)	C11—C12—C13	130.7 (4)
N1—C1—O1	112.8 (4)	C11—C12—H12	114.7
N1—C1—C10	129.0 (4)	C13—C12—H12	114.7
O1—C1—C10	118.1 (3)	C12—C11—S2	123.7 (3)
C4—N3—S1	131.0 (3)	C12—C11—H11	118.1
C4—N3—H3	114.5	S2—C11—H11	118.1
S1—N3—H3	114.5	C4—C5—C6	120.2 (4)
C2—N2—N1	105.8 (3)	C4—C5—H5	119.9
C1—C10—S2	114.5 (3)	C6—C5—H5	119.9
C1—C10—H10A	108.6	N2—C2—O1	113.3 (4)
S2—C10—H10A	108.6	N2—C2—C3	129.0 (4)
C1—C10—H10B	108.6	O1—C2—C3	117.7 (4)
S2—C10—H10B	108.6	C5—C4—C9	118.9 (4)
H10A—C10—H10B	107.6	C5—C4—N3	124.0 (4)
C14—C13—C18	117.8 (4)	C9—C4—N3	117.1 (4)
C14—C13—C12	120.1 (4)	C16—C17—C18	121.5 (5)
C18—C13—C12	122.0 (4)	C16—C17—H17	119.3
C15—C14—C13	121.6 (5)	C18—C17—H17	119.3
C15—C14—H14	119.2	C16—C15—C14	119.5 (5)
C13—C14—H14	119.2	C16—C15—H15	120.2
C6—C7—C8	121.5 (4)	C14—C15—H15	120.2
C6—C7—Cl	119.3 (4)	C17—C16—C15	119.5 (5)
C8—C7—Cl	119.2 (4)	C17—C16—H16	120.3
C7—C6—C5	119.6 (4)	C15—C16—H16	120.3
C7—C6—H6	120.2		
			
N2—N1—C1—O1	−1.1 (4)	O2—S1—C3—C2	178.8 (3)
N2—N1—C1—C10	175.3 (4)	N3—S1—C3—C2	−69.4 (4)
C2—O1—C1—N1	0.9 (4)	C14—C13—C12—C11	138.1 (6)
C2—O1—C1—C10	−175.9 (3)	C18—C13—C12—C11	−44.4 (8)
O3—S1—N3—C4	−28.4 (5)	C13—C12—C11—S2	−6.0 (9)
O3—S1—N3—C4	−28.4 (5)	O4—S2—C11—C12	89.9 (5)
O2—S1—N3—C4	−159.1 (4)	O5—S2—C11—C12	−41.7 (5)
C3—S1—N3—C4	89.3 (5)	C10—S2—C11—C12	−154.9 (5)
C1—N1—N2—C2	0.8 (4)	C7—C6—C5—C4	1.1 (7)
N1—C1—C10—S2	108.0 (4)	N1—N2—C2—O1	−0.2 (5)
O1—C1—C10—S2	−75.8 (4)	N1—N2—C2—C3	−178.2 (4)
O4—S2—C10—C1	48.6 (3)	C1—O1—C2—N2	−0.4 (4)
O5—S2—C10—C1	175.8 (3)	C1—O1—C2—C3	177.8 (3)
C11—S2—C10—C1	−69.1 (3)	S1—C3—C2—N2	−76.1 (6)
C18—C13—C14—C15	2.5 (7)	S1—C3—C2—O1	106.1 (4)
C12—C13—C14—C15	−179.9 (5)	C6—C5—C4—C9	−3.4 (7)
C8—C7—C6—C5	2.4 (7)	C6—C5—C4—N3	177.6 (4)
Cl—C7—C6—C5	−177.2 (4)	C8—C9—C4—C5	2.3 (7)
C6—C7—C8—C9	−3.5 (7)	C8—C9—C4—N3	−178.6 (4)
Cl—C7—C8—C9	176.2 (4)	S1—N3—C4—C5	−2.1 (7)
C7—C8—C9—C4	1.1 (7)	S1—N3—C4—C9	178.9 (3)
C14—C13—C18—C17	−1.8 (7)	C13—C18—C17—C16	−0.3 (7)
C12—C13—C18—C17	−179.3 (4)	C13—C14—C15—C16	−1.1 (8)
O3—S1—C3—C2	49.3 (4)	C18—C17—C16—C15	1.8 (8)
O3—S1—C3—C2	49.3 (4)	C14—C15—C16—C17	−1.0 (8)

### Hydrogen-bond geometry (Å, º)

Cg is the centroid of the C4–C9 ring.

**Table:**

*D*—H···*A*	*D*—H	H···*A*	*D*···*A*	*D*—H···*A*
C5—H5···O3	0.93	2.41	3.010 (6)	122
N3—H3···O5^i^	0.86	2.19	2.900 (5)	140
C3—H3*B*···O2^ii^	0.97	2.38	3.198 (5)	141
C6—H6···O4^iii^	0.93	2.45	3.290 (5)	151
C12—H12···O5^iii^	0.93	2.60	3.242 (5)	127
C14—H14···*Cg*^iv^	0.93	2.90	3.670 (5)	141

Symmetry codes: (i) *x*, −*y*−1/2, *z*+1/2; (ii) −*x*, −*y*, −*z*+2; (iii) *x*, *y*+1, *z*; (iv) *x*, −*y*+1/2, *z*−1/2.
